# Overburden in Caregivers of Chronically Ill Adolescents: A Comparative Study between Pulmonological and Endocrinological Diseases

**DOI:** 10.3390/children10121840

**Published:** 2023-11-23

**Authors:** Elvira García-Marín, Selene Valero-Moreno, Konstanze Schoeps, Silvia Postigo-Zegarra, Marián Pérez-Marín

**Affiliations:** 1 Personality, Assessment and Psychological Treatment Department, Faculty of Psychology and Speech Therapy, University of Valencia, Avenida Blasco Ibáñez, 21, 46010 Valencia, Spain; elgarma2@alumni.uv.es (E.G.-M.); konstanze.schoeps@uv.es (K.S.); silvia.postigo@universidadeuropea.es (S.P.-Z.); marian.perez@uv.es (M.P.-M.); 2 Faculty of Health Sciences, Department of Psychology, European University of Valencia, Passeig. de l’Albereda, 7, 46010 Valencia, Spain

**Keywords:** disease, pediatric chronic disease, family, family system, psychological impact, caregiver, respiratory disease, endocrinological disease

## Abstract

Chronic pediatric disease has a major impact on the life of a child and his or her family. In this sense, the figure of the main caregiver is relevant, who may present adjustment difficulties before the disease, accompanied by high levels of stress and emotional discomfort, which interfere with the adolescent’s adjustment before the new situation. The aim of this research was to carry out a comparative study among caregivers of adolescents with various diseases, analyzing the risk and protective factors for the stress presented by this population. For this purpose, a sample of 406 main caregivers of adolescents with an endocrinological or pneumological disease from different hospitals in Valencia was used, where characteristics related to the disease, attachment, type of family, and emotional distress of these caregivers were considered. In general, the results show higher levels of stress in caregivers of adolescents with an endocrinological disease, specifically DM1, and found significant predictors of anxiety-depressive symptomatology, the need for approval, and several variables related to the disease. These data reveal the importance of providing comprehensive care to the family system, offering health skills to overcome diseases, and reinforcing the protective factors offered by the family system.

## 1. Introduction

Chronic illness (CI) during adolescence profoundly impacts the lives of the afflicted individuals and their families. The diagnosis of CI is often accompanied by a series of adjustments in response to a condition that will persist with the child or adolescent for an extended period. In this context, CI refers to a condition with a prolonged course, typically necessitating continuous medical attention [1,2]. Its incidence in childhood is steadily increasing, affecting 10–30% of the pediatric population presently [3].

In this regard, a diverse range of chronic illnesses affect patients and their families differently. Among them are respiratory diseases, which impact the organs constituting the respiratory system and are vital for breathing. Many pediatric respiratory diseases exist, with asthma being the most prevalent. In Spain, 10% of children under 14 years of age are afflicted with this condition [4]. Bronchial asthma frequently co-occurs with another increasingly prevalent condition, allergies. The World Health Organization (WHO) has classified allergies among the six most common diseases worldwide, affecting around 30% of the global population and nearly 80% of families experiencing some form of allergy [5].

Apart from asthma and allergies, other conditions related to the endocrine system are also prevalent during this stage [6]. These include type 1 diabetes mellitus (T1DM) [6] and short stature (SS). Data indicate that SS is more prevalent in girls, primarily in 13-year-old patients [7], with an estimated occurrence of 24.90% to 26.20% in pediatric patients [8]. On the other hand, the global prevalence of T1DM ranges from 0.8 to 4.6 per 1000 individuals [9], with the highest incidence occurring during childhood, typically between 10 and 14 years of age [10].

Caregivers play a crucial role, particularly in the context of illness. A caregiver is someone who provides support to those in need. Adolescents with CI require specialized long-term assistance and ongoing treatment from caregivers [3]. The burden shouldered by these individuals elicits a complex response, encompassing emotional, physical, and social changes, as well as other adverse consequences [11,12].

Faced with this new situation, the families of adolescents with CI undergo moments of insecurity and uncertainty, grappling with inevitable adaptive and emotional changes [13]. These families experience elevated stress levels and intense emotional strain related to the illness, surpassing the stress levels observed in parents of healthy children [14]. The literature indicates that parents of adolescents with T1DM exhibit higher stress levels, followed by those of children facing respiratory issues, with caregivers of adolescents with short stature displaying lower stress levels [13].

Moreover, a strong correlation exists between stress and emotional disorders. The literature reveals elevated levels of anxiety and depression among caregivers of chronically ill individuals compared to parents of healthy children [15], along with increased stress and family dysfunction [16]. Primary caregivers also grapple with various social consequences, including stigmatization, which further heightens anxiety, stress, and depression [17]. 

Additionally, it is important to highlight the significance of parenting styles and family bonds. As per [18], parenting style refers to the parent-child relationship in everyday life. Thus, the mental health of parents can have a significant influence on their children. It has been observed that children of parents with emotional disorders exhibit lower levels of resilience in the face of adversity, as well as poorer mental and physical health. Conversely, positive family relationships and parenting styles result in better adaptation and increased resilience [19]. Studies suggest that families characterized by strong cohesion and flexibility employ more coping strategies and possess better self-efficacy while also demonstrating lower levels of stress and family conflicts [20]. Therefore, families with pediatric patients with chronic illnesses report higher conflict levels than families with healthy children [21].

Based on these data, it is evident that caregivers of adolescents with chronic illness may encounter elevated stress levels, along with symptoms of both anxiety and depression, contributing to increased family dysfunction. These aspects vary depending on the type of illness, but there is still a lack of comparative studies demonstrating these differences. Thus, this study aimed to conduct a comparative analysis among caregivers of adolescents with different illnesses through examining the risk and protective factors related to the stress experienced by this population. Three hypotheses were proposed in relation to the stated objective:

**Hypothesis 1 (H1):**  *Caregivers of adolescents with an endocrinological illness will exhibit higher levels of stress and emotional distress than caregivers of those with a pneumological illness. Additionally, caregivers with more dysfunctional family types and insecure attachments will manifest heightened stress levels*.

**Hypothesis 2 (H2):**  *Stress will exhibit a positive correlation with emotional distress and a negative correlation with characteristics associated with secure attachment or a more functional family system*.

**Hypothesis 3 (H3):**  *Stress levels will be negatively influenced by protective factors, such as secure attachment and a functional family, and positively influenced by risk factors, including emotional distress*.

## 2. Materials and Methods

### 2.1. Participants and Setting

The sample for this study comprised 406 primary caregivers of adolescents with CI, drawn from the allergy, pneumology, and endocrinology units of several hospitals, using a cross-sectional study design with incidental sampling. Of these, 61.8% of the pediatric patients were male (n = 251), while 38.2% were female (n = 155), with ages ranging from 12 to 17 years (M = 13.59; SD = 1.277). Through choosing a 95% confidence level and a sample size of 406 participants, the margin of error was ±4%, which means that the study had enough participants to achieve sufficient statistical power. Among them, 39.7% had an endocrinological illness (n = 161) and 60.3% had a pneumoallergic illness (n = 245). Concerning the primary caregivers, 82.70% were mothers, 15.30% were fathers, and the remaining had a different relationship with the patient. Their average age was 45.68 years, with ages ranging from 31 to 63 years. Moreover, 76.5% were married (n = 299), 15.8% were separated (n = 62), while the remainder had a different marital status.

Data were collected from various hospitals and assessments were conducted at a single time point. This was a convenience sample, which took into account a number of inclusion and exclusion criteria. Information was gathered through interviews with primary caregivers, with the assistance of healthcare professionals. The inclusion criteria for adolescents included ages between 12 and 17 years and a confirmed diagnosis of an endocrinological or pneumoallergic illness lasting at least 6 months. For caregiver participants, the inclusion criteria mandated that they were the primary caregivers of the adolescents. The exclusion criteria encompassed adolescents with additional physical or psychological conditions and caregivers who did not serve as primary caregivers. 

### 2.2. Ethical Considerations

This work complies with the ethical criteria of the Declaration of Helsinki (2013) and has the endorsement of the Ethics Committee of the corresponding institutions (UV-INV_ETICA-1226194).

### 2.3. Variables and Instruments

Demographic information on the primary caregivers was collected, including age, employment status, marital status, and gender. Additionally, various variables related to the adolescents were recorded, such as the type of illness, time since diagnosis and treatment, hospital admissions, visit frequency, and the average duration of hospital stay.

The Pediatric Inventory for Parents (PIP, [22]) was employed to assess the perceived stress level in relation to the illness. This questionnaire evaluates the stress experienced by parents caring for children with chronic illnesses. It has previously been utilized in studies involving parents of children facing various conditions, including type 1 diabetes mellitus [23] and cancer [24]. The questionnaire presents various highly stressful situations that these parents encounter, measuring both the effort required and the frequency of these situations. This study utilized a condensed version of the original questionnaire [13], comprising 12 Likert-scale items. The frequency dimension demonstrated a reliability of 0.81, while the effort dimension exhibited a reliability of 0.74.

Anxiety-depressive symptoms and emotional distress were measured using the Hospital Anxiety and Depression Scale (HADS–[25]). This instrument, consisting of 14 items, includes two subscales (HAD-A for anxiety and HAD-D for depression) rated on a Likert scale. Reliability indices for primary caregiver family members were 0.74 for the anxiety dimension, 0.77 for the depression scale, and 0.83 for emotional distress.

The Family Cohesion and Adaptability Scale (CAF, FACES III [26]) examined family cohesion and adaptability. This scale, designed to evaluate variables affecting family dynamics, comprises 20 items rated on a Likert scale. It yields six subscales, which, when combined, result in the dimensions of family cohesion and adaptability. Lastly, three family types were identified: balanced, mid-range, or unbalanced. This instrument is based on a circumplex model; therefore, reliability calculations are not applicable.

Finally, attachment was assessed using the Adult Attachment Questionnaire (CAA, [27]), a 40-item Likert-scale instrument. It encompasses various aspects related to attachment style, categorized into four dimensions: the need for approval, hostile conflict resolution, ease of expressing feelings, and emotional self-sufficiency.

### 2.4. Data Analysis

To conduct the corresponding analyses, the statistical software SPSS v.28 was utilized, and the following tests were performed: descriptive statistics (frequencies, percentages, mean, and standard deviation), chi-square (χ2), one-way ANOVA to compare means based on the type of illness, Pearson’s correlation coefficient to analyze the relationship between study variables, and hierarchical multiple regression (HMR) in steps to predict perceived stress levels based on the type of illness and protective and risk factors.

## 3. Results

### 3.1. Mean Comparisons Based on the Type of Illness

Stress levels were compared based on the type of illness. Significant differences were noted between the two types of illnesses, with higher scores in both frequency and effort observed in the case of endocrinological illnesses (Table 1).

Regarding symptoms of anxiety and depression, no statistically significant differences were observed based on the type of illness. However, distinctions were noted in overall emotional distress, with higher emotional distress reported in the case of endocrinological illnesses. Regarding attachment, no significant differences were found in the various dimensions based on the type of illness. In general, it was observed that in both groups, primary caregivers displayed a low need for approval, moderately hostile conflict resolution, moderate emotional expression, and a high degree of emotional self-sufficiency. The results for each dimension were similar for both illness groups.

There were no significant differences in family cohesion based on the type of illness, with an isolated family type predominating in both cases. No differences were observed in adaptability, and structured and flexible adaptations were predominant in both cases. As for family type, no differences were observed, with balanced families predominating in both cases.

### 3.2. Relationship between the Study Variables

The correlation between stress and various illness-related, family, and psychological variables was examined. A moderate positive correlation was found between the frequency and effort of the stressors. Concerning illness-related variables, very low positive correlations were observed between stress (both effort and frequency) and the number of hospitalizations, average duration of hospital stay, treatment duration, and time since diagnosis. However, a very low negative correlation was noted between the frequency dimension of stress and the time since diagnosis.

In terms of psychological and family variables, both the frequency and effort of stress showed a low positive correlation with anxiety, depression, and the need for approval. They also had very low positive correlations with conflict resolution and emotional self-sufficiency. Additionally, the effort dimension exhibited a low negative correlation with the age of the primary caregiver and a moderate positive correlation with emotional distress (Table 2).

### 3.3. Hierarchical Regression Models

Subsequently, the predictive capacity of the studied variables was assessed using hierarchical regression. The criterion variables comprised the frequency and effort dimensions of stress, while the predictor variables encompassed the type of illness, illness-related factors, attachment variables, family-related factors, and symptoms of anxiety and depression. Both models consisted of six steps: the first involved the type of illness, the second incorporated illness-related factors, the third introduced attachment variables, the fourth included family-related characteristics, and the fifth and sixth integrated anxiety and depression. To perform the linear regression analysis, five key assumptions were addressed: First, linearity was assessed through generating a scatter plot of unstandardized predicted values versus unstandardized residuals, revealing a low positive correlation. Next, the independence of errors was evaluated using the Durbin–Watson statistic, and the results fell within the range of 1.5 to 2.5, supporting the assumption of independence. Homoscedasticity was confirmed through a scatter plot examination, demonstrating consistent variance in the residuals, thus validating this assumption. Furthermore, normality of residuals was examined via a histogram and a normal probability plot, revealing a normal distribution and affirming the normality assumption. Lastly, a test for non-collinearity was conducted, resulting in Variance Inflation Factor (VIF) scores below 10, and an analysis of tolerance indicated the absence of collinearity. With all these assumptions met, the linear regression model was subsequently executed.

Regarding the frequency dimension of stress, it was noted that the type of illness significantly increased the variance by 2.6% in the first step (ΔR^2^ = 0.026; *p* = 0.005). In the second step, illness-related factors increased the variance by 3.3% (ΔR^2^ = 0.033; *p* = 0.016). The third step, introducing attachment-related factors, contributed to a 6.6% increase in the variance (ΔR^2^ = 0.066; *p* ≤ 0.001). The fourth step, involving family adaptability and cohesion, did not significantly increase the variance (ΔR^2^ = 0.004; *p* = 0.529). In the fifth step, depressive symptoms accounted for a 7.1% increase in the variance (ΔR^2^ = 0.071; *p* ≤ 0.001). Finally, anxiety symptoms contributed to a 1.9% increase in the variance (ΔR^2^ = 0.019; *p* = 0.007). Consequently, it was observed that having an endocrinological illness (β = 0.127), a higher number of previous hospitalizations (β = 0.124), a greater need for approval (β = 0.135), and increased symptoms of anxiety and depression (β_anxiety_ = 0.175; β_depression =_ 0.227) were associated with a higher frequency of stress (Table 3).

For the effort dimension of stress, it was observed that the type of illness significantly increased the variance by 4.1% in the first step (ΔR^2^ = 0.041, *p* ≤ 0.001). In the second step, illness-related variables did not contribute to an increase in the variance (ΔR^2^ = 0.022, *p* = 0.072). In the third step, attachment-related variables led to a notable 10.1% increase in the variance (ΔR^2^ = 0.101, *p* ≤ 0.001). The fourth step, involving family adaptability and cohesion, did not yield a significant increase in the variance (ΔR^2^ = 0.002, *p* = 0.756). Moving to the fifth step, the presence of depressive symptoms accounted for a 5.3% increase in the variance (ΔR^2^ = 0.053, *p* ≤ 0.001). Lastly, the emergence of anxiety symptoms contributed to a 1.2% increase in the variance (ΔR^2^ = 0.012, *p* = 0.030).

In this context, having an endocrinological illness (β = 0.179), a higher number of previous hospitalizations (β = 0.122), a greater need for approval (β = 0.162), and increased symptoms of anxiety and depression (β_anxiety_ = 0.140; β_depression_ = 0.199) were linked to a heightened perception of stress within the effort dimension.

## 4. Discussion

The impact of chronic illness during a developmental stage, such as adolescence, increases the perceived stress on the family system, requiring continuous adjustments and adaptations to cope with the various challenges associated with this stage and the illness [3,11,12]. Numerous studies have addressed the impact of chronic illnesses on the family system, both in Spain [11,14] and worldwide [28,29,30]. Nevertheless, comparative studies based on the type of illness are limited, as they have primarily centered on contrasting caregivers of healthy adolescents with those of adolescents with illnesses [14]. 

Nonetheless, this subject is especially pertinent due to the growing prevalence of these diseases in children and adolescents. The aim of this research was to assess the levels of perceived stress and emotional distress among caregivers, considering the type of disease (endocrinological and pneumoallergic) and other variables related to the family system, such as attachment and family characteristics.

Based on the results obtained and the hypotheses put forward, significant differences in stress and emotional distress were expected to be found in caregivers depending on the type of disease, with those with endocrinological diseases perceiving the worst emotional adjustment and the highest levels of stress (Hypothesis 1), in line with previous studies [12,13]. The results are along these lines, showing that caregivers of patients with endocrinological diseases experience higher levels of stress, both in frequency and strain, along with greater emotional distress, compared to those caring for pneumological diseases. However, because of the limited number of studies available, further research is required to investigate whether additional variables, such as disease severity, may also be impacted.

In line with previous studies suggesting that the diagnosis of a chronic illness can increase the number of family conflicts and potentially disrupt the family system [20,21], it was anticipated that the group of caregivers facing higher levels of stress and distress, particularly those dealing with endocrinological diseases, would exhibit more dysfunctional characteristics. However, our findings revealed similar family characteristics, characterized by isolated cohesion and structured and flexible adaptability, and no significant differences were observed.

Although we did not find prior studies comparing attachment and family types based on the type of illness, our results do not align with the existing literature, which suggests that families with pediatric patients tend to report higher family conflict compared to families with healthy children [21]. This unexpected outcome may be explained by the observed similarities, in line with other research indicating that strong family cohesion and greater flexibility serve as protective factors against elevated stress levels and family conflicts related to illness [20]. As a result, further research is needed to comprehensively investigate the relationship between these variables and caregivers’ perceived stress levels.

In the second part of this study, we expected to find positive associations between perceived stress and emotional distress (Hypothesis 2), as indicated in previous studies [15,16,17]. Additionally, it was anticipated that stress would positively correlate with disease-specific variables, such as the duration of diagnosis, hospitalizations, or treatment duration [13]. Previous research has shown that these variables were positively related [2]. Our results align with those of prior research, indicating that caregivers with higher levels of perceived stress are also associated with increased symptoms of anxiety and depression. Continuous exposure to caregiving-related situations, such as emergency room visits or extended treatment periods, may lead to an elevation in perceived stress.

Regarding family and psychological variables, it was observed that stress in terms of effort and frequency was positively correlated with the symptoms of anxiety and depression, the need for approval, and hostile conflict resolution. Conversely, it was negatively correlated with the expression of positive feelings and the age of the pediatric patient. These correlations are consistent with the existing literature, suggesting that stress levels are positively associated with emotional distress [15], dysfunctional family functioning [20,21], and adverse events that negatively impact attachment [28]. However, it is once again emphasized that more studies addressing family variables are needed, as they have not been extensively explored in previous research.

Finally, through prediction models, Hypothesis 3 aimed to assess whether there were differences in perceived stress in terms of frequency (how often one is exposed) and effort (the burden it places on the caregiver), taking into account risk factors (need for approval, anxiety and depression, and hostile conflict resolution) and protective factors (expression of feelings and functional family cohesion and adaptability). It also aimed to evaluate whether the type of illness and its associated variables were predictors of perceived stress levels. The results reveal that higher levels of perceived stress in both dimensions are positively associated with the number of hospitalizations, the need for approval, and the symptoms of anxiety and depression. Once again, the type of illness, specifically endocrinological, appears to positively correlate with perceived stress levels. All of this aligns with previous studies [16,28]. This study suggests that the type of illness, particularly endocrinological diseases, is strongly associated with higher levels of perceived stress. However, it is important to emphasize the need for further research in this direction.

Although the data obtained provide relevant information about the impact of diseases on caregivers, this study has several limitations. Firstly, only a limited number of diseases were studied, which, despite being the most common in Spain, do not represent the varied situations of other diseases. Additionally, this research was conducted in different hospitals in a specific region, which may limit the generalizability of the results to other autonomous communities. Regarding the study sample, most participants were women, with mothers generally responsible for the care of chronically ill children, and previous studies have confirmed that it is frequently mothers who are the main carers. The use of self-reports may incur social desirability bias, so it would be necessary to incorporate other informants, such as the patients themselves and qualitative information to understand the experience of caregiving more broadly. Finally, one limitation is that the participants were caregivers of a characteristic developmental stage, such as adolescence, so these results cannot be generalized to caregivers of other developmental stages, such as childhood. It would be interesting to address the possible differences in future research. In future studies, it is advisable to expand the number of pathologies studied, diversify the sample, and include other psychological and emotional disorders, among other factors.

## 5. Conclusions

In conclusion, the practical implications of this study highlight the need for the development of clinical intervention programs aimed at improving the well-being of family members caring for chronically ill individuals. These programs should focus on teaching resilient coping strategies for handling stressful situations, ultimately minimizing the physical and psychological impact on caregivers. To accomplish this, a multidisciplinary approach to managing chronic illnesses is crucial, with a particular focus on the inclusion of psychology professionals. These professionals play a vital role in supporting individuals and families throughout a chronic illness through promoting adaptive coping behaviors, ensuring treatment adherence, and providing guidance on various social and medical aspects. Furthermore, it is imperative for state policies to recognize and support the essential role of caregivers in the healthcare system. State governments should allocate resources to develop and implement programs that specifically target the well-being of caregivers, providing them with the necessary tools and support to navigate the challenges of caring for chronically ill individuals. These policies can encompass financial assistance, respite care services, and educational resources for caregivers, all of which can contribute to their overall well-being and the quality of care provided to those with chronic illnesses.

Ultimately, acknowledging the central role that primary caregivers play in the healthcare of pediatric patients underscores the significance of addressing both the physical and psychological well-being of these caregivers. This entails promoting healthy adaptive behaviors in response to illness and strengthening the protective elements within the family structure, supported through well-designed state policies and programs aimed at caregiver support.

## Figures and Tables

**Table 1:** Mean comparison according to the type of illness for all study variables.

Variable	Type of Illness								*F*	*p*	*η* ^2^
	Pneumological Illness				Endocrinological Illness						
	*M*	*SD*			*M*	*SD*					
Stress frequency	2.759	0.685			2.995	0.745			10.768	0.001	0.026
Stress effort	2.035	0.731			2.310	0.066			12.277	0.001	0.030
	Absence n (%)	Possible n (%)	Presence n (%)		Absence n (%)	Possible n (%)	Presence n (%)	*χ*	$\chi^{2}$	*p*	*V*
Anxiety symptomatology	135 57.0%	62 26.2%	40 16.9%		84 53.2%	35 22.2%	39 24.7%		3.755	0.153	0.098
Depressive symptomatology	206 86.9%	25 10.5%	6 2.5%		130 82.3%	47 11.9%	12 3.0%		1.648	0.439	0.065
Emotional distress	220 93.6%		15 6.4%		138 87.3%		20 12.7%		4.586	0.032	0.108
	Low score n (%)	Medium score n (%)	High score n (%)		Low score n (%)	Medium score n (%)	High score n (%)		$\chi^{2}$	*p*	*V*
Need for approval	96 45.1%	83 39.0%	34 16.0%		59 39.1%	71 47.0%	21 13.9%		2.348	0.309	0.080
Hostile conflict resolution	80 36.7%	122 56.0%	16 7.3%		45 29.4%	98 64.1%	10 6.5%		2.491	0.288	0.082
Emotional expression	36 17.4%	102 49.3%	69 33.3%		27 18.2%	68 48.6%	45 32.1%		0.210	0.901	0.025
Emotional self-sufficiency	103 46.6%	93 42.1%	25 11.3%		73 47.7%	56 36.6%	24 15.7%		2.025	0.363	0.074
	Uninterested n (%)	Isolated n (%)	United n (%)	Interlaced n (%)	Uninterested n (%)	Isolated n (%)	United n (%)	Interlaced n (%)	$\chi^{2}$	*p*	*V*
Family cohesion	46 19.7%	94 40.3%	76 32.6%	17 7.3%	40 25.3%	62 39.2%	49 31.0%	7 4.4%	2.694	0.441	0.083
	Rigid	Structured	Flexible	Chaotic	Rigid	Structured	Flexible	Chaotic			
Family adaptability	6 2.5%	119 50.4%	102 43.2%	9 3.8%	6 3.8%	91 57.2%	60 37.7%	2 1.3%	4.227	0.238	0.103
	Dysfunctional	Intermediate	Balanced		Dysfunctional	Intermediate	Balanced				
Family type	6 2.6%	66 28.4%	160 69.0%		0 0.0%	50 31.8%	107 68.2%		4.432	0.109	0.107

**Table 2:** Relationship between stress and the different variables (n = 406).

	1	2	3	4	5	6	7	8	9	10	11	12	13	14	15	16
Stress effort	1															
Stress frequency	0.468 **	1														
Adolescent age	−0.058	−0.075	1													
Diagnosis time	0.096	−0.025	0.230 **	1												
Treatment time	0.075	0.035	0.219 **	0.684 **	1											
Hospitalizations	0.151 **	0.122 *	0.043	0.260 **	0.290 **	1										
Average stay length	0.101	0.057	−0.156	0.067	−0.040	0.294 **	1									
Caregiver age	−0.208 **	−0.067	0.218 **	0.068	−0.016	−0.077	−0.034	1								
Anxiety	0.371 **	0.344 **	0.019	0.051	0.074	0.015	0.023	−0.058	0.006	1						
Depression	0.343 **	0.305 **	0.022	0.024	0.066	0.022	0.029	−0.070	−0.019	0.560 **	1					
Emotional distress	0.410 **	0.367 **	0.024	0.044	0.081	0.020	0.030	−0.071	−0.006	0.904 **	0.860 **	1				
Need for approval	0.300 *	0.315 **	0.140 **	0.004	0.013	−0.003	0.032	−0.053	0.022	0.466 **	0.428 **	0.506 **	1			
Hostile conflict resolution	0.114 *	0.117 *	0.089	−0.033	−0.003	−0.057	−0.096	−0.066	0.050	0.193 **	0.259 **	0.251 **	0.483 **	1		
Emotional expression	−0.077	−0.152 **	−0.022	−0.040	−0.018	0.050	0.021	−0.054	0.027	−0.187 **	−0.289 **	−0.261 **	−0.244 **	−0.206 **	1	
Emotional self-sufficiency	0.091	0.082	0.085	0.053	0.005	0.000	0.048	0.25	−0.053	0.119 *	0.131 **	0.141 **	0.311 **	0.336 **	−0.234 **	1

r = Pearson’s r. * *p ≤* 0.05; ** *p ≤* 0.01.

**Table 3:** Regression models for predicting stress (frequency and effort).

Predictor	Stress Frequency					Stress Effort				
	***R* ^2^ *corr***	**Δ*R*^2^**	**Δ*F***	**β**	***t***	***R* ^2^ *corr***	**Δ*R*^2^**	**Δ*F***	**β**	***t***
Step 1	0.022 **	0.026 **	7.991			0.038 ***	0.041 ***	13.091		
Type of illness				0.160 **	2.827				0.203 ***	3.618
Step 2	0.046 **	0.033 **	3.488			0.051	0.022	2.351		
Type of illness				0.160 **	2.858				0.204 ***	3.639
Diagnosis time				0.136	1.708				−0.080	−1.001
Treatment time				−0.055	−0.677				0.113	1.407
Hospitalizations				0.128 **	2.188				0.114	1.947
Step 3	0.103 ***	0.066 ***	7.458			0.145 ***	0.101 ***	12.053		
Type of illness				0.154 **	2.828				0.198 ***	3.718
Diagnosis time				0.136	1.754				−0.083	−1.091
Treatment time				−0.056	−0.715				0.109	1.426
Hospitalizations				0.130	2.266				0.125	2.232
Need for approval				0.276 ***	4.200				0.281	4.378
Hostile conflict resolution				−0.059	−0.919				−0.069	−1.104
Emotional expression				−0.018	−0.313				−0.134	−2.352
Step 4	0.101	0.004	0.637			0.141	0.002	0.280		
Type of illness				0.149 **	2.728				0.197 ***	3.681
Diagnosis time				0.136	1.744				−0.085	−1.120
Treatment time				−0.051	−0.646				0.110	1.438
Hospitalizations				0.128 **	2.224				0.125 **	2.227
Need for approval				0.278 ***	4.224				0.282 ***	4.387
Hostile conflict resolution				−0.063	−0.982				−0.071	−1.128
Emotional expression				−0.014	−0.243				−0.133 **	−2.338
Family cohesion				−0.057	−1.033				−0.033	−0.620
Family adaptability				0.031	0.561				−0.019	−0.353
Step 5	0.172 ***	0.071 ***	26.418			0.192 ***	0.053 ***	19.849		
Type of illness				0.135 **	2.571				0.185 ***	3.562
Diagnosis time				0.147 *	1.968				−0.075	−1.022
Treatment time				−0.078	−1.038				0.087	1.186
Hospitalizations				0.123 **	2.229				0.121 **	2.220
Need for approval				0.174 ***	2.633				0.194 ***	2.962
Resolución Hostile conflict resolution				−0.066	−1.065				−0.073	−1.200
Emotional expression				0.062	1.075				−0.068	−1.188
Family cohesion				−0.016	−0.301				0.001	0.027
Family adaptability				0.045	0.849				−0.007	−0.134
Depresión				0.311 ***	5.140				0.266 ***	4.455
Step 6	0.189 **	0.019 **	7.266			0.202 **	0.012 **	4.735		
Type of illness				0.127 **	2.434				0.179 ***	3.454
Diagnosis time				0.135	1.825				−0.085	−1.154
Treatment time				−0.076	−1.022				0.088	1.189
Hospitalizations				0.124 **	2.270				0.122 **	2.250
Need for approval				0.135 **	2.008				0.162 **	2.439
Resolución Hostile conflict resolution				−0.062	−1.014				−0.070	−1.156
Emotional expression				0.062	1.076				−0.069	−1.206
Family cohesion				−0.009	−0.177				0.007	0.127
Family adaptability				0.042	0.799				−0.010	−0.184
Depression				0.227 ***	3.369				0.199 **	2.977
Anxiety				0.175 ***	2.696				0.140 **	2.176
Durbin–Watson	1.844					1.975				

*R*^2^ = change in *R*^2^; *F* = change in *F*; β = regression coefficient; *t* = *t*-statistic value; * *p* ≤ 0.05. ** *p* ≤ 0.01. *** *p* ≤ 0.001.

## Data Availability

The data presented in this study are available on request from the corresponding author. The data are not publicly available due to the data privacy and confidentiality.
